# Core-Shell Sr_2_CeO_4_@SiO_2_ Filled COC-Based Composites with Low Dielectric Loss for High-Frequency Substrates

**DOI:** 10.3390/polym13224006

**Published:** 2021-11-19

**Authors:** Qinlong Wang, Hao Wang, Caixia Zhang, Qilong Zhang, Hui Yang

**Affiliations:** 1 State Key Laboratory Silicon Mat, School of Materials Science and Engineering, Zhejiang University, Hangzhou 310027, China; 21926093@zju.edu.cn (Q.W.); ustbwanghao@163.com (H.W.); yanghui@zju.edu.cn (H.Y.); 2 Jiaxing Glead Elect Co., Ltd., Jiaxing 314003, China; z363238730@163.com

**Keywords:** polymer composite, dielectric loss, thermal conductivity, stability

## Abstract

High-frequency communication equipment urgently needs substrate materials with lower dielectric loss, better heat dissipation, and higher stability, to ensure real-time low-loss and high-speed signal transmission. The core-shell structure of Sr_2_CeO_4_@SiO_2_ was prepared by the sol-gel method, and the modified powders with different volume contents were introduced into the cyclic olefin copolymer (COC) to prepare hydrocarbon resin-based composites. Due to the protective effect of the SiO_2_ shell, the stability of the powders is significantly improved, and the moisture barrier and corrosion resistance of the composites are enhanced, which is conducive to the normal operation of electronic equipment in harsh and complex environments. When the filler content is 20 *vol*%, the composite has a dielectric loss of 0.0023 at 10 GHz, a dielectric constant of 3.5, a thermal conductivity of 0.9 W·m^−1^·K^−1^, a water absorption of 0.32% and a coefficient of thermal expansion of 37.7 ppm/°C. The COC/Sr_2_CeO_4_@SiO_2_ composites exhibit excellent dielectric properties and thermal conductivity, while maintaining good moisture resistance and dimensional stability, which shows potential application prospects in the field of high-frequency substrates.

## 1. Introduction

In recent years, 5G communication technology has rapidly developed at an unexpected rate. This fast and convenient new-generation communication technology can achieve faster signal transmission at millimeter wave frequencies, and plays an important role in instant messaging, unmanned driving, mobile computing, and other fields. The signal transmission at a high frequency means that there are higher requirements for the properties of substrate materials, such as lower dielectric loss, higher thermal conductivity and lower water absorption [1,2]. With the integration and miniaturization of electronic equipment, electronic components will face a more complex external environment. As a carrier to protect and support electronic components, substrate materials need to be improved in terms of their stability and safety [3,4,5,6]. The preparation of substrate materials with an excellent comprehensive performance has become the focus of the 5G communications industry.

Among the low-ε_r_ and low-τ_ε_ high-frequency substrates, the resin materials with good application prospects mainly include polytetrafluoroethylene (PTFE) [7,8], modified polypheylene ether (MPPE) [9,10], modified polyimide (MPI) [11], liquid crystal polymer (LCP) [12,13], etc. As an important engineering plastic, cyclic olefin copolymer (COC) is mainly used in optics, packaging materials, biomedicine, electronic components, and other fields [14,15]. Considering the excellent properties of cyclic olefin copolymer (COC), such as an extremely low dielectric constant and dielectric loss (ε_r_ = 2.2, τ_ε_ = 0.0002 at 10 GHz), low coefficients of thermal expansion (CTE, 60 ppm/°C) and low water absorption (<0.1%), we use cyclic olefin copolymer (COC) as the resin matrix to explore its possible applications in the high-frequency field.

In previous reports, the introduction of inorganic fillers into the resin matrix, such as BN [16,17,18], Si_3_N_4_ [19], SiO_2_ [20], Al_2_O_3_ [21], AlN [22], and SiC [23], has become a common method to improve the performance of substrate materials. However, the presence of different functional groups on the surface of the filler results in a poor compatibility between the filler and the polymer. Therefore, inorganic fillers need to be modified using appropriate methods to improve the interface bonding with the matrix. As a multiphase structure of the core and the outer shell, the core-shell structure has an ideal modification effect. A variety of core-shell structures were prepared, including AgNWs@SiO_2_ [24], Al@SiO_2_ [25], BN@AgNPs [26], and introduced into the polymer matrix to improve the performance of composites. In our previous work, Sr_2_CeO_4_ with excellent properties (ε_r_ = 14.49, Q × f = 120,886 GHz) was prepared by solid-phase sintering [27]. However, when exposed to the air for a long time, the powders tend to absorb water and form agglomerates. We prepared Sr_2_CeO_4_@SiO_2_ core-shell structure using the sol-gel method. The coated SiO_2_ enhanced the stability of the powders and promoted the dispersion of the powders into the polymer.

In this work, we used COC as the resin matrix and modified Sr_2_CeO_4_@SiO_2_ as the ceramic fillers to prepare composites with excellent comprehensive properties. First, the core-shell structure of Sr_2_CeO_4_@SiO_2_ was prepared using a simple sol-gel method. Then, the polymer and the fillers were uniformly mixed in an organic solvent to prepare the raw material. Finally, the substrate material was prepared by hot pressing and laminated with copper foil to obtain a copper-clad laminate. The dielectric properties, thermal conductivity, stability and other composite properties with different volume contents of Sr_2_CeO_4_@SiO_2_ were studied.

## 2. Materials and Methods

### 2.1. Materials

SrCO_3_ was purchased from Macleans Biochemical Technology Co., Ltd., Shanghai, China. CeO_2_ was purchased from Aladdin Biochemical Technology Co., Ltd., Shanghai, China. Ethyl orthosilicate (TEOS), cyclohexane (C_6_H_12_) and ammonium hydroxide (NH_4_OH, 28 wt.%) were purchased from Sinopharm Group Chemical Reagent Co., Ltd., Shanghai, China. Cyclic olefin copolymer (COC, 6017) was purchased from TOPAS Advanced Polymers Co., Ltd., Frankfurt, Germany.

### 2.2. Synthesis and Surface Modification of Sr_2_CeO_4_

The pure-phase Sr_2_CeO_4_ was prepared by the solid-phase sintering method. First, high-purity SrCO_3_ and CeO_2_ were uniformly mixed by ball-milling at a molar ratio of 2:1, and calcined at 1150 °C for 4 h to obtain a preliminary calcined Sr_2_CeO_4_. Then, the powders were dispersed in ethanol and ball-milled at a speed of 300 r/min for 12 h. After being sufficiently crushed, the powders were sintered again at 1150 °C for 4 h to ensure that the reaction was complete. Finally, the powders were ball-milled twice and dried to obtain pure-phase Sr_2_CeO_4_.

**Scheme 1:**
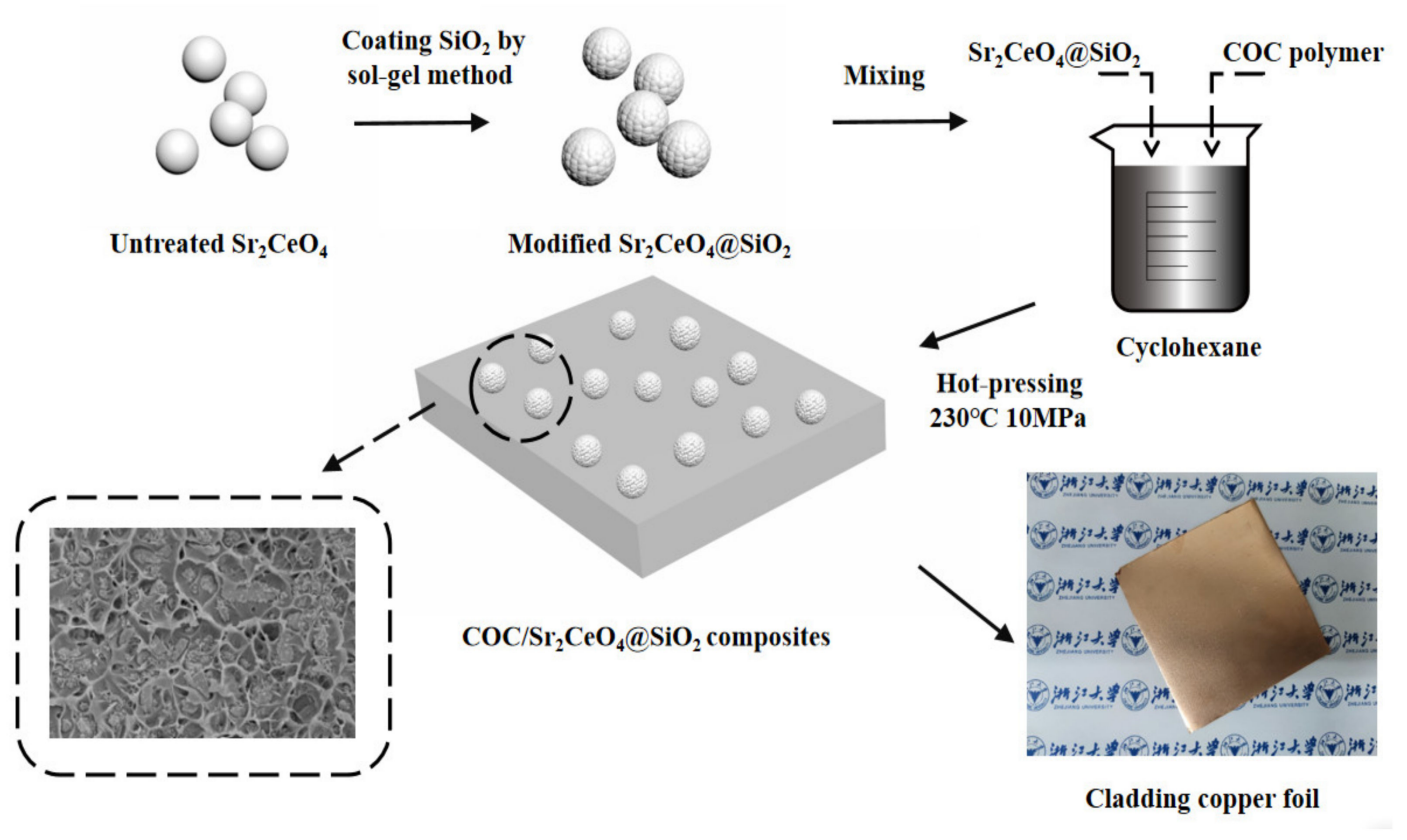
The fabrication process of COC/Sr_2_CeO_4_@SiO_2_ composites.

As shown in Scheme 1, the core-shell structure of Sr_2_CeO_4_@SiO_2_ was prepared by the sol-gel method. First, 5 g Sr_2_CeO_4_ was added to 50 mL ethanol, and the mixture was stirred to completely disperse the powders. Then, 200 mL ethanol, 90 mL deionized water, and 10 mL NH_4_OH were added to the mixture. After a thorough mixing, 10 mL TEOS ethanol solution (1.5 *vol*%) was added dropwise at a constant speed and stirred at room temperature for 12 h to fully react. The molar ratio of Sr_2_CeO_4_ to TEOS in the mixture was 19.7:1. Finally, the resulting product was centrifuged at 8000 rpm for 3 min and washed twice with ethanol.

### 2.3. Fabrication of COC/Sr_2_CeO_4_@SiO_2_ Composites

The COC/Sr_2_CeO_4_@SiO_2_ composites with different filler contents were fabricated. First, M_1_ g Sr_2_CeO_4_@SiO_2_ filler was uniformly dispersed in cyclohexane under ultrasonic treatment. Then, M_2_ g COC polymer was dissolved in cyclohexane, added to the dispersion and stirred for 10 h. The mixture was dried at 80 °C for 6 h to remove residual cyclohexane. Finally, the raw materials were hot-pressed at 230 °C and 5 MPa for 1 h to obtain COC/Sr_2_CeO_4_@SiO_2_ composites. The total volume V_t_ of the sample was determined by calculating the volume of the hot-pressing mold. The density of COC polymer was recorded as ρ_2_ (1.02 g/cm^3^). The density of untreated Sr_2_CeO_4_ is 5.4 g/cm^3^, and the density of modified Sr_2_CeO_4_@SiO_2_ were recorded as ρ_1_, which can be calculated by the following formula:

(1) $$\rho_{1}=\frac{M_{1}}{V_{t}-M_{2}/\rho_{2}}$$

The volume content of Sr_2_CeO_4_@SiO_2_ in the composites was recorded as *φ*, which can be calculated by the following formula:

(2) $$\varphi =\frac{M_{1}/\rho_{1}}{V_{t}}\times 100\%$$

Unmodified COC/Sr_2_CeO_4_ composites were fabricated under the same conditions for comparison. The COC/Sr_2_CeO_4_@SiO_2_ composite with the best properties was laminated with copper foil at 230 °C, 5 MPa and −0.085 MPa for 0.5 h to prepare copper-clad laminate. To prevent oxidation of the copper foil, a vacuum environment was maintained during the preparation process.

### 2.4. Characterization

The micro-morphology and structure of fillers and composites were observed on a scanning electron microscope (FESEM, SU-8010, Hitachi Ltd., Tokyo, Japan). The core-shell structure and element distribution of Sr_2_CeO_4_@SiO_2_ were observed on a transmission electron microscope (TEM, HT-7700, Hitachi Ltd., Tokyo, Japan) with Cu K_α_ radiation. The laser particle size analyzer (LS13320, Beckman Coulter, California, USA) was used to measure the particle size distribution of Sr_2_CeO_4_ and Sr_2_CeO_4_@SiO_2_. The contact-angle-measuring device (OCA 20, Dataphysics Co., Stuttgart, Germany) was used to measure the contact angle of Sr_2_CeO_4_ and Sr_2_CeO_4_@SiO_2_. The chemical composition of the filler was characterized by X-ray diffraction (XRD, EMPYREAN, PANalytical Co., Almelo, Netherlands), and X-ray photoelectron spectroscopy (XPS, K-Alpha, Thermo Fisher, MA, USA). XPS test used Al K_α_ (1486.6 eV) radiation source at room temperature, and the vacuum degree of the analysis chamber was maintain above 5.0 × 10^−^^7^ mBar. The pass energy of full-spectrum scan and narrow-spectrum scan were 100 eV and 50 eV, respectively. The resolution of the instrument was determined to be 0.8 ~ 1.0 eV through the full width at half maximum. The charge correction of the test result was carried out, with C 1s = 284.80 eV as the energy standard. The high-frequency dielectric performance at 10 GHz was tested by Split-Cylinder Resonator (QWED), and the low-frequency dielectric performance at 10^4^ Hz–10^6^ Hz was tested by impedance analyzer (TH2839). The thermal conductivity was tested by thermal conductivity tester (TPS 2500 S, Hot Disk, Uppsala, Sweden). The temperature changes in the composites under heating and cooling were recorded by a thermal infrared camera (H13, HIKMICRO, Hangzhou, China). The water absorption of the composites was tested according to IPC-TM-650 2.6.2.1. The initial mass of the sample was measured in advance. The sample was then placed in a container of distilled water and kept at a temperature of 23 ± 1.1 °C for 24 h. After wiping off the surface moisture, the quality of the sample was measured again. The water absorption was obtained by calculating the ratio of the increased mass to the initial mass. The corrosion resistance was characterized by observing the surface of the samples. The samples were placed in 2 mol/L hydrochloric acid, sodium hydroxide, and isopropanol solutions for 5 min each. The surface morphology of the samples before and after corrosion were observed by scanning electron microscope. According to IPC-TM-650 2.4.41, the coefficient of thermal expansion of the samples was tested by a thermomechanical analyzer (TA Q400, TA Instruments, PA, USA). According to IPC-TM-650 2.4.4B, the flexure strength of the samples was measured by a universal material testing machine (CMT5205).

## 3. Result and Discussion

### 3.1. Characterization of Fillers and Composites

**Figure 1:**
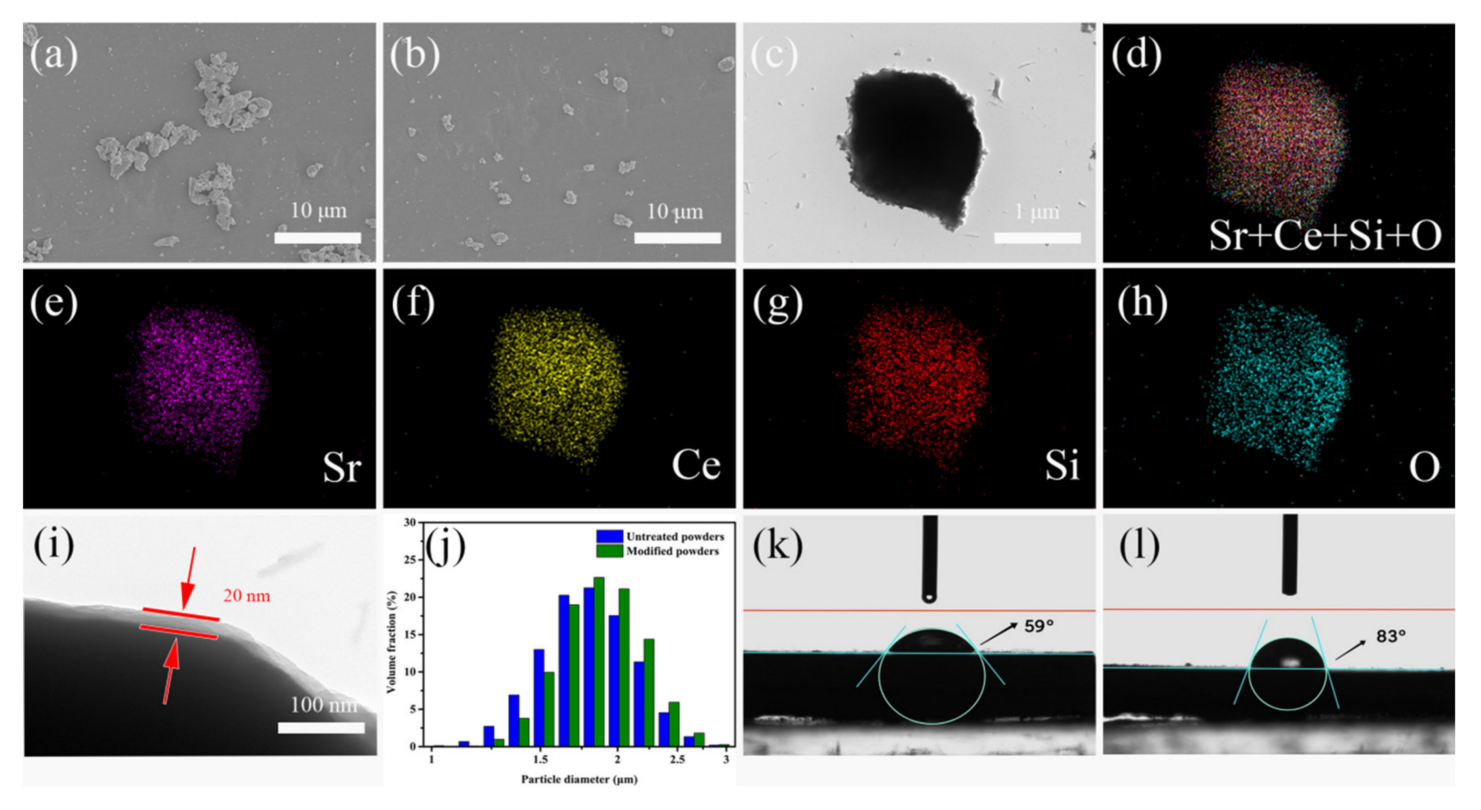
(**a**) SEM image of untreated Sr_2_CeO_4_; (**b**) modified Sr_2_CeO_4_@SiO_2_; (**c**) TEM image of modified Sr_2_CeO_4_@SiO_2_; (**d**–**h**) corresponding electron microscopy images of modified Sr_2_CeO_4_@SiO_2_; (**i**) TEM image of SiO_2_ shell; (**j**) the particle size distribution; (**k**) the hydrophobic angle of untreated Sr_2_CeO_4_; (**l**) modified Sr_2_CeO_4_@SiO_2_.

Figure 1a,b shows the morphology of untreated Sr_2_CeO_4_ and modified Sr_2_CeO_4_@SiO_2_. The untreated Sr_2_CeO_4_ have obvious agglomeration, due to the stronger interaction force between the particles. Due to the presence of the coated SiO_2_ shell, the modified Sr_2_CeO_4_@SiO_2_ has better dispersion characteristics. In the TEM image of Sr_2_CeO_4_@SiO_2_ shown in Figure 1c, we can see that the particle has an obvious core-shell structure. To further verify the composition of the coating, an energy spectrum analysis was performed on the particle. Figure 1d–h shows the electron microscope images of Sr_2_CeO_4_@SiO_2_. The elements of the particle are composed of Sr, Ce, Si and O. By observing the distribution of single elements, we can see that Sr and Ce are distributed in the core of the particle, and Si and O are distributed in the outer shell of the particle. Due to the coating of the SiO_2_ shell on the particle surface, the distribution range of Si and O is wider than that of Sr and Ce. The partially enlarged TEM image of the core-shell structure (Figure 1i) shows that the thickness of the SiO_2_ shell is about 20 nm. The formation of amorphous SiO_2_ is due to the successive hydrolysis and condensation of TEOS in solution [28]:

Hydrolysis reaction: Si(OR)_4_ + xH_2_O = Si(OR)_4 − x_(OH)_x_ + xROH (3)

Condensation reaction: ≡Si–OH + HO–Si≡ = ≡Si–O–Si≡ + H_2_O (4)

≡Si–OH + RO–Si≡ = ≡Si–O–Si≡ + ROH(5)

The particle size distribution is shown in Figure 1j. The average particle sizes of Sr_2_CeO_4_ and Sr_2_CeO_4_@SiO_2_ were 1.82 μm and 1.89 μm, respectively. Figure 1k,l shows the comparison of hydrophobic angles between Sr_2_CeO_4_ and Sr_2_CeO_4_@SiO_2_. The hydrophobic angle of Sr_2_CeO_4_ increased from 59° to 83° after surface modification. This evidence can also supplement the successful coating of the SiO_2_ shell.

**Figure 2:**
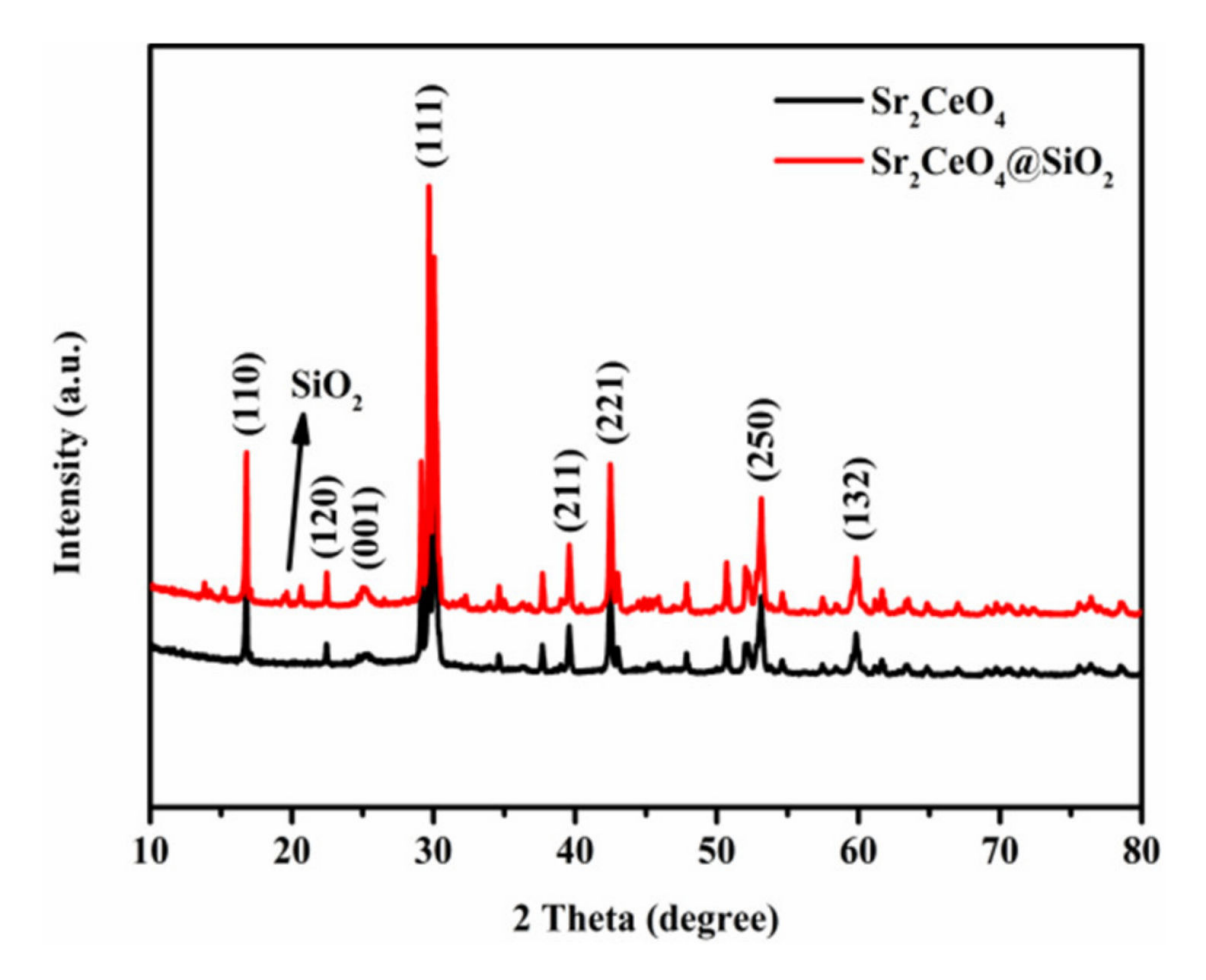
XRD patterns of Sr_2_CeO_4_ and Sr_2_CeO_4_@SiO_2_.

**Figure 3:**
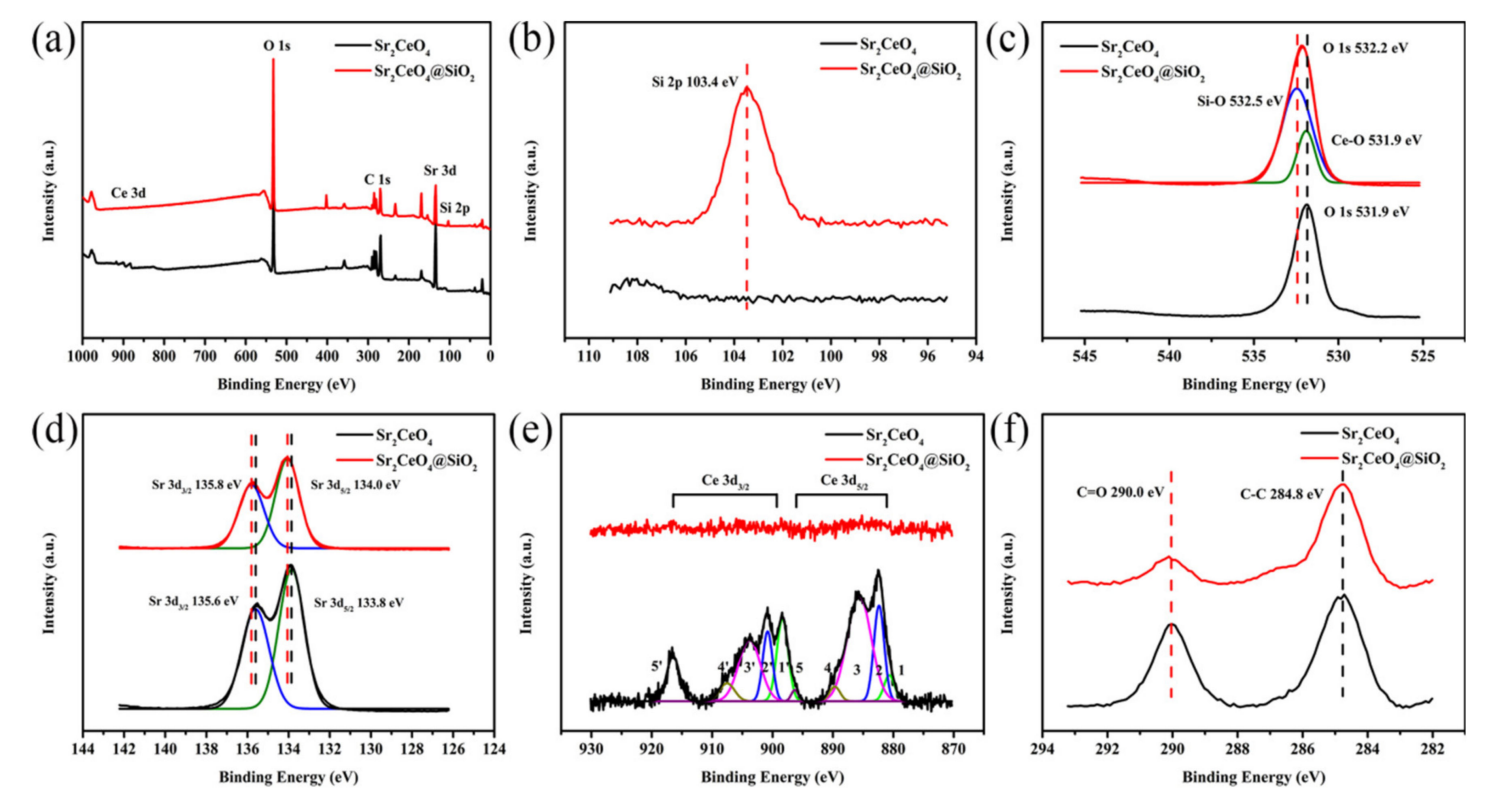
(**a**) XPS survey spectra of full spectrum; (**b**) Si 2p; (**c**) O 1s; (**d**) Sr 3d; (**e**) Ce 3d; (**f**) C 1s spectrum of Sr_2_CeO_4_ and Sr_2_CeO_4_@SiO_2_.

In the XRD diffraction pattern (Figure 2), the characteristic peaks in Sr_2_CeO_4_ and Sr_2_CeO_4_@SiO_2_ are basically the same. The characteristic peaks at 14.2°, 27.9°, 37.7°, 42.5°, 52.0°, 59.9° correspond to the (110), (111), (211), (221), (250), (132) crystal planes. For modified powders, the diffraction pattern has a very weak diffraction peak at 20°–30°, which mainly corresponds to the existence of amorphous SiO_2_. The composition of the coating layer is further verified by the XPS spectrum in Figure 3a. Both samples show characteristic peaks in Sr 3d, Ce 3d, O 1s in the XPS full spectrum. The characteristic peak in Si 2p appears in the XPS spectrum of Sr_2_CeO_4_@SiO_2_, which further confirms the composition of the coating layer [29,30]. Figure 3b–f shows the high-resolution spectra of Si 2p, O 1s, Sr 3d, Ce 3d, and C 1s for the two systems, respectively. Table 1 shows the chemical composition of the samples obtained by the XPS method. Figure 3b shows that the Si 2p spectrum only has a characteristic peak at 103.4 eV in the modified powders. In Figure 3c, the peak in O 1s in the modified powders has shifted by 0.3 eV compared to the untreated powders, which can be attributed to the presence of the SiO_2_ shell. The O 1s signal in the modified powders mainly comes from the Si-O bond of the outer shell, but also contains part of the Ce-O bond of the inner core [31]. As shown in Figure 3d, the two peaks in Sr 3d are located at 134.0 eV (Sr 3d_5/2_) and 135.8 eV (Sr 3d_3/2_) in the modified powders, and the two peaks are separated by 1.8 eV spin orbits. Compared with the untreated powders, the binding energy of Sr 3d in the modified powders increased by 0.2 eV, indicating that Sr lost electrons and transferred them to the SiO_2_ shell. Moreover, the peak intensity of Sr 3d in the modified powders is weaker than that of the untreated powders due to the presence of the SiO_2_ shell. The thickness of the SiO_2_ shell in Figure 1i is about 20 nm, and the concentration of Ce 3d in the modified powders in Table 1 is only 0.16%, resulting in no obvious characteristic peaks of Ce 3d in the modified powders (Figure 3e). The peaks in Ce 3d are observed in the untreated powders, and both the Ce 3d_3/2_ and Ce 3d_5/2_ peaks have complex internal structures [32]. The ten peaks corresponding to the five pairs of spin–orbit doublets are displayed by fitting. Peaks 2 (2′), 4 (4′) and 5 (5′) are located at 882.4 eV (900.8 eV), 889.7 eV (907.5 eV), and 896.3 eV (916.5 eV), respectively, corresponding to Ce^4+^ from Sr_2_CeO_4_. Peaks 1 (1′) and 3 (3′) are located at 880.7 eV (898.4 eV) and 885.7 eV (903.8 eV) respectively, corresponding to Ce^3+^ oxides from the surface [33,34]. As shown in Figure 3f, the peaks in the C 1s at 284.8 eV and 290.0 eV correspond to C–C and C=O, respectively, which originate from the surface contamination of the samples. Based on the above characterization, it can be proved that the surface of Sr_2_CeO_4_ particles is coated with amorphous SiO_2_.

**Figure 4:**
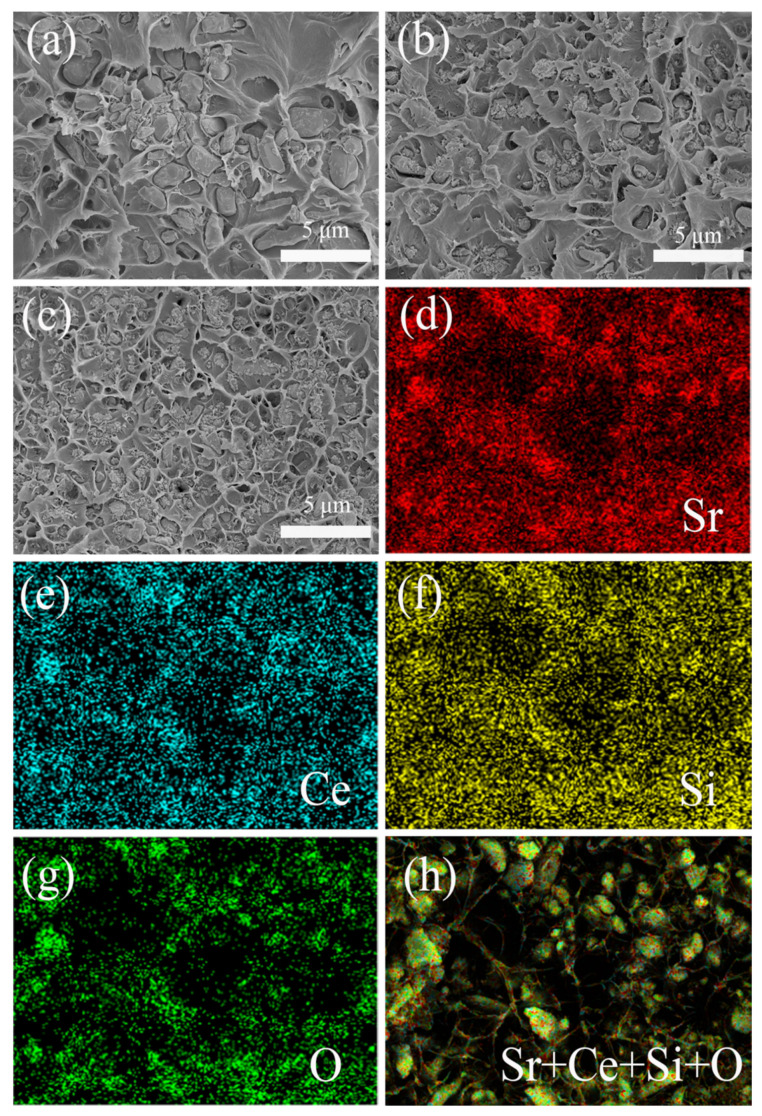
Cross-sectional SEM images of composites with different filler content: (**a**) COC/10 *vol*% Sr_2_CeO_4_; (**b**) COC/10 *vol*% Sr_2_CeO_4_@SiO_2_; (**c**) COC/20 *vol*% Sr_2_CeO_4_@SiO_2_; (**d**–**h**) corresponding electron microscope images of COC/20 *vol*% Sr_2_CeO_4_@SiO_2_.

Figure 4a,b shows the cross-sectional SEM images of the composites filled with 10 *vol*% untreated powders and modified powders. Due to the existence of the SiO_2_ shell, the modified powders are evenly distributed in the polymer, while the untreated powders are more prone to agglomeration. Figure 4c shows the cross-sectional SEM image of COC/20 *vol*% Sr_2_CeO_4_@SiO_2_. At a higher filler content, the modified powders still maintain a uniform distribution. Figure 4d–h shows electron microscope images based on Figure 4c. Although the fillers are randomly distributed in the polymer, the distribution of different elements is relatively uniform, which further verifies the dispersion characteristics of the modified fillers. In addition, due to the uneven morphology of Sr_2_CeO_4_, a small amount of SiO_2_ microspheres were produced during the coating process. For the coating of irregular particles, the existing sol-gel process needs to be further optimized.

### 3.2. Dielectric Properties

**Figure 5:**
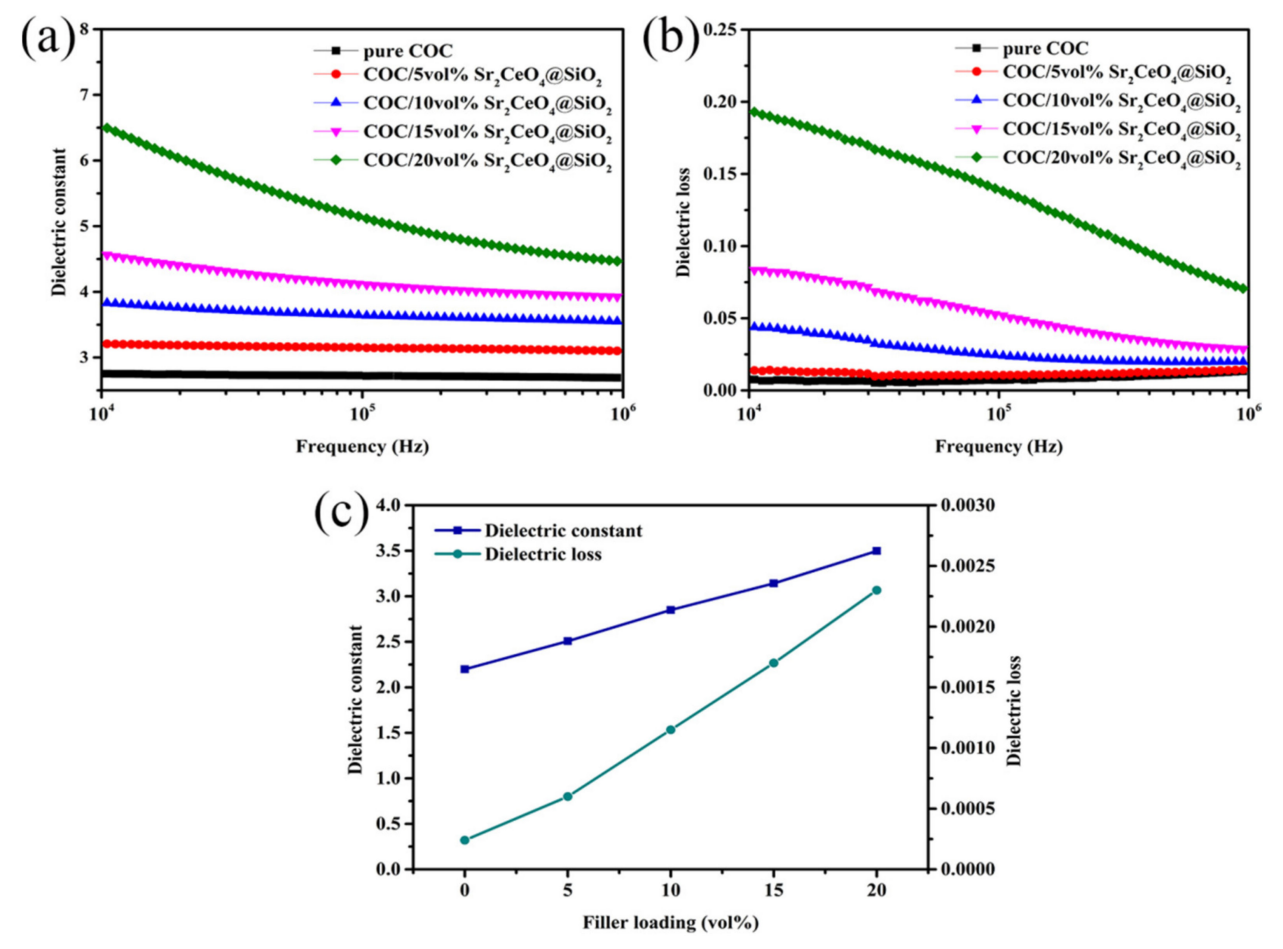
(**a**,**b**) The dielectric constant and dielectric loss of COC/Sr_2_CeO_4_@SiO_2_ composites in range of 10^4^–10^6^ Hz; (**c**) the dielectric constant and dielectric loss of COC/Sr_2_CeO_4_@SiO_2_ composites under 10 GHz.

Figure 5a–c shows the dielectric properties of the COC/Sr_2_CeO_4_@SiO_2_ composites at different frequencies. Since the dielectric constant (ε_r_) of the filler is higher than that of pure COC, the ε_r_ of the composites at a high frequency (10 GHz) and low frequency (10^4^–10^6^ Hz) increases with the introduction of fillers. The ε_r_ of COC/20 *vol*% Sr_2_CeO_4_@SiO_2_ at 10 GHz is 3.5, while the ε_r_ of pure COC is 2.2. The polarization mechanisms in composites include electronic polarization, ion polarization and orientation polarization, and different polarization mechanisms have different frequency responses [8,35]. The dielectric constant of composites is affected by multiple polarization mechanisms. As the frequency continues to increase, the oriented polarization effect gradually weakens, so the ε_r_ of the composites continues to decrease. When the frequency rises to 10 GHz, the oriented polarization effect disappears, so the ε_r_ at high frequencies (10 GHz) is lower than that at low frequencies (10^4^–10^6^ Hz). The ε_r_ of COC/20 *vol*% Sr_2_CeO_4_@SiO_2_ at 10 GHz is 3.5, which contrasts with the ε_r_ of 6.5 at 10^4^ Hz.

Since Sr_2_CeO_4_@SiO_2_ has a higher dielectric loss (τ_ε_) compared to the COC matrix, the τ_ε_ of the composites increases with the introduction of fillers. As the frequency increases, the orientation polarization gradually weakens until it disappears [30], resulting in a decrease in the τ_ε_ of the composites. When the filler content is 20 *vol*%, the τ_ε_ of the composite is 0.11 at 10^6^ Hz and decreases to 0.0023 at 10 GHz, which shows that the τ_ε_ at high frequencies is significantly lower than that at low frequencies. In summary, the COC-based composite material filled with Sr_2_CeO_4_@SiO_2_ has lower dielectric constant (<3.5) and lower dielectric loss (<0.0023) at high frequencies, which can meet the dielectric performance requirements of high-frequency substrates.

### 3.3. Thermal Conductivity

**Figure 6:**
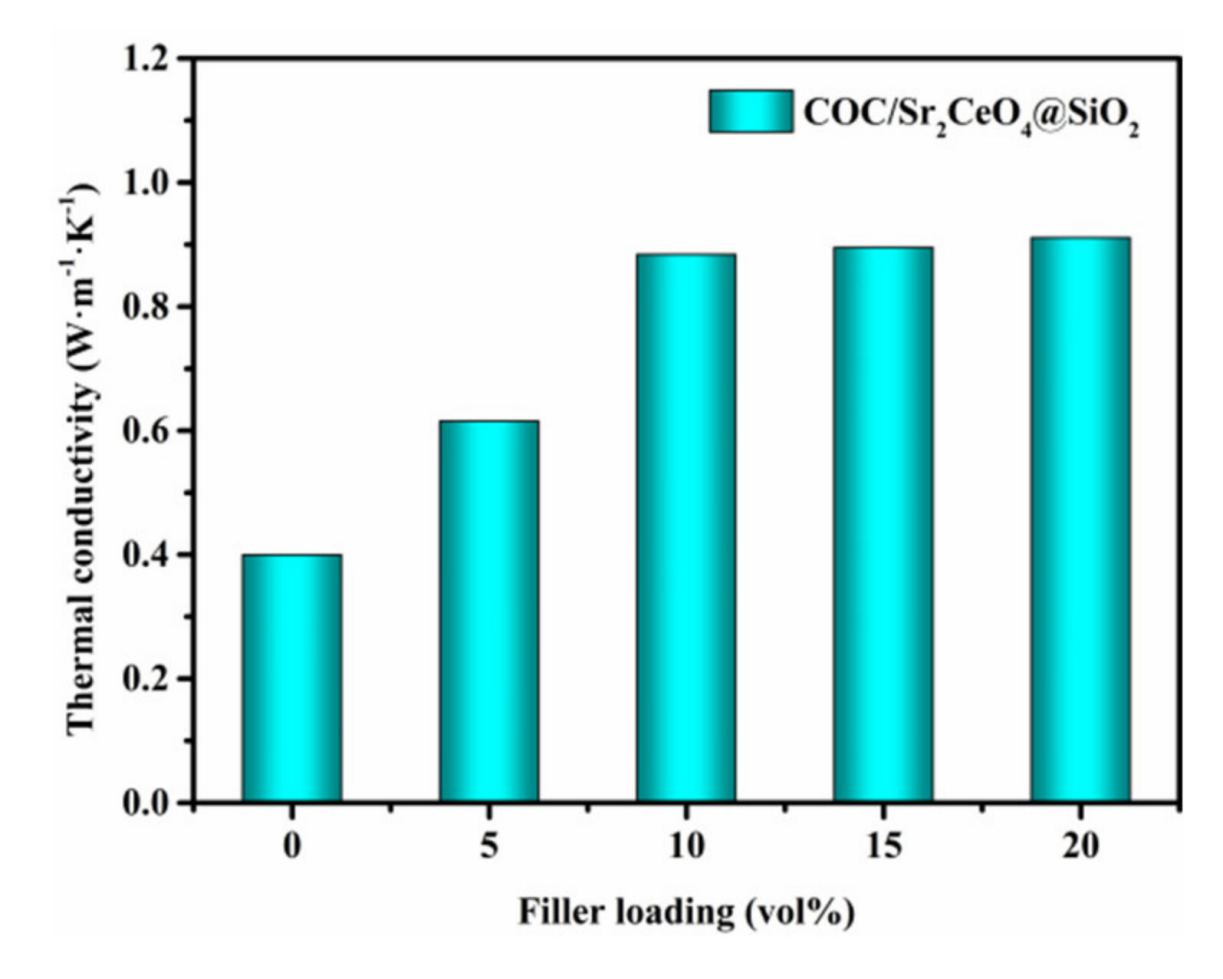
The in-plane thermal conductivity of COC/Sr_2_CeO_4_@SiO_2_ composites.

Figure 6 shows the thermal conductivity (TC) of the COC/Sr_2_CeO_4_@SiO_2_ composites. As the filler content increases, the TC of the composites continues to increase, reaching 0.91 W m^−1^ K^−1^ at a filler content of 20 *vol*%. However, it is worth noting that the rate of increase in TC is variable. When the filler loading is low (≤10 *vol*%), the TC of the composites increases significantly. With the further increase in filler loading (>10 *vol*%), the TC of the composites slowly changes, which can be attributed to the limited thermal conductivity of the filler and the increase in the interface thermal resistance between the filler and the matrix [36]. In addition, the introduction of more defects will also have an adverse effect on heat transfer [37].

**Figure 7:**
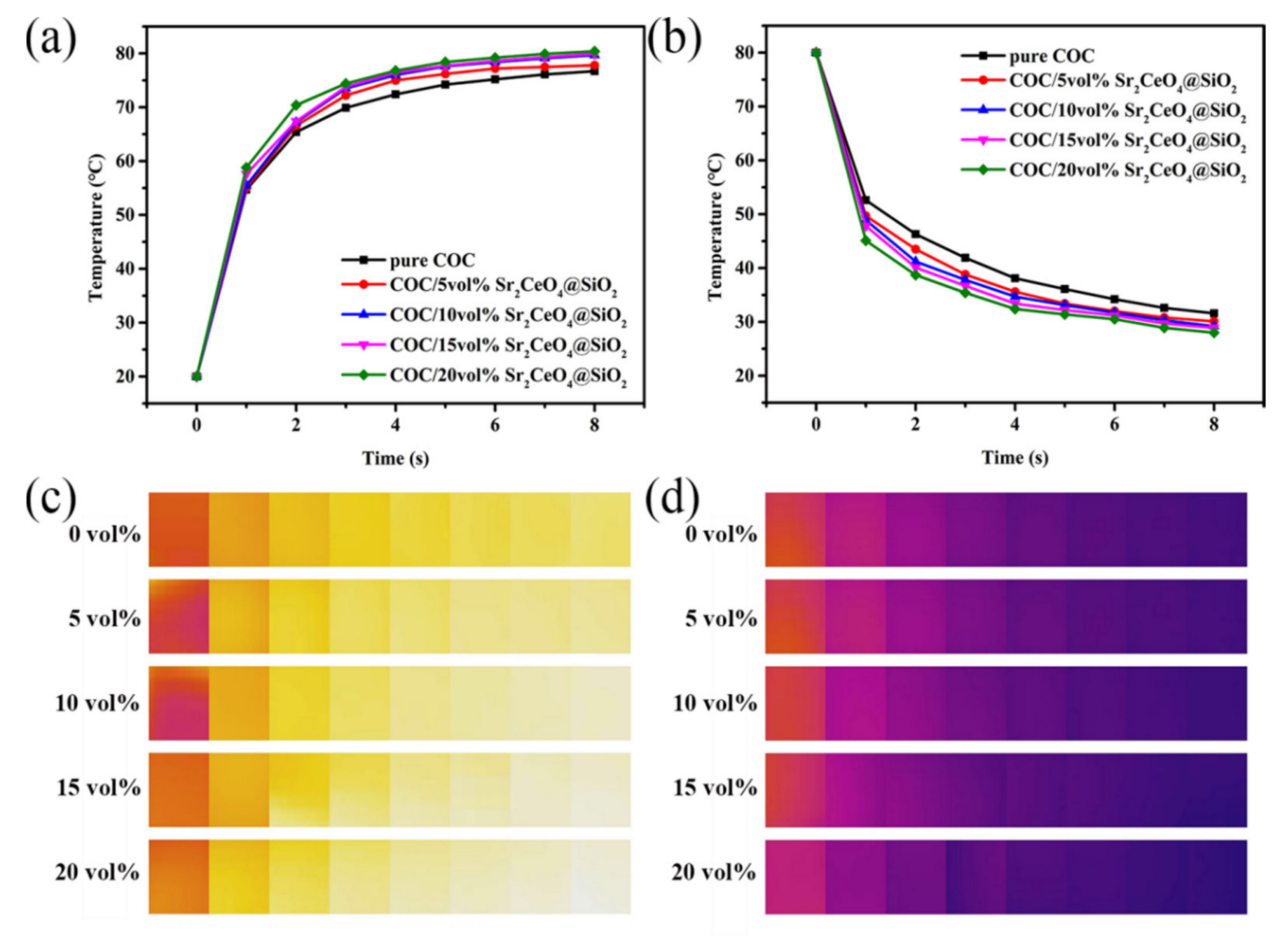
The average temperature change in the samples (**a**) during the heating process; (**b**) during the cooling process; the thermal infrared imaging of the samples (**c**) during the heating process; (**d**) during the cooling process.

To verify the TC of the composites, the samples were characterized by infrared thermal imaging. The samples were cut into 2 cm × 2 cm squares in advance, and the temperature of the heating stage was kept at 80 °C. The cut sample was quickly placed on the heating stage, and the heating process of the sample was recorded with a thermal imager. When the sample temperature rose to 80 °C, the sample was quickly transferred to the normal-temperature desktop, and the temperature change during the cooling process was recorded again. Figure 7 shows the temperature change curve and thermal infrared image of the heating and cooling process. The introduction of filler leads to an increase in the heating and cooling rate of the samples. In summary, the filler Sr_2_CeO_4_@SiO_2_ helps to improve the heat transfer capability of the composites.

### 3.4. Moisture Absorption and Corrosion Resistance

**Figure 8:**
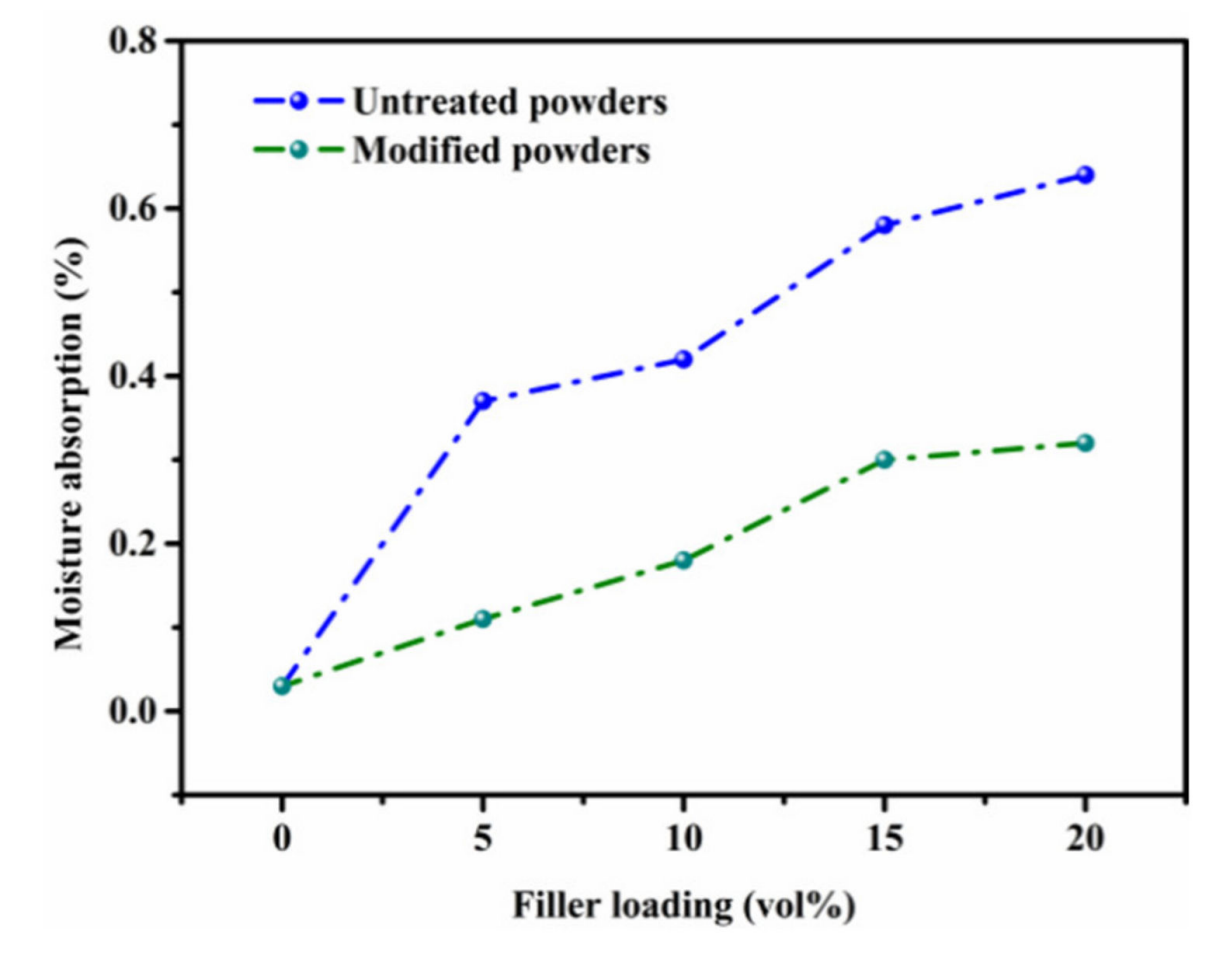
The water absorption of COC/Sr_2_CeO_4_ and COC/Sr_2_CeO_4_@SiO_2_ composites.

Untreated Sr_2_CeO_4_ has a certain degree of water absorption and will easily condense into lumps when exposed to the air for a long time. To reduce the water absorption of the composites, a SiO_2_ shell was coated on the surface of the particle. Figure 8 shows the water absorption of the composites before and after modification. Due to the protective effect of the SiO_2_ shell, the water absorption of the modified composites is reduced to less than 0.32%. In addition, the modified powders have good hydrophobicity, which is also conducive to the improvement in stability.

**Figure 9:**
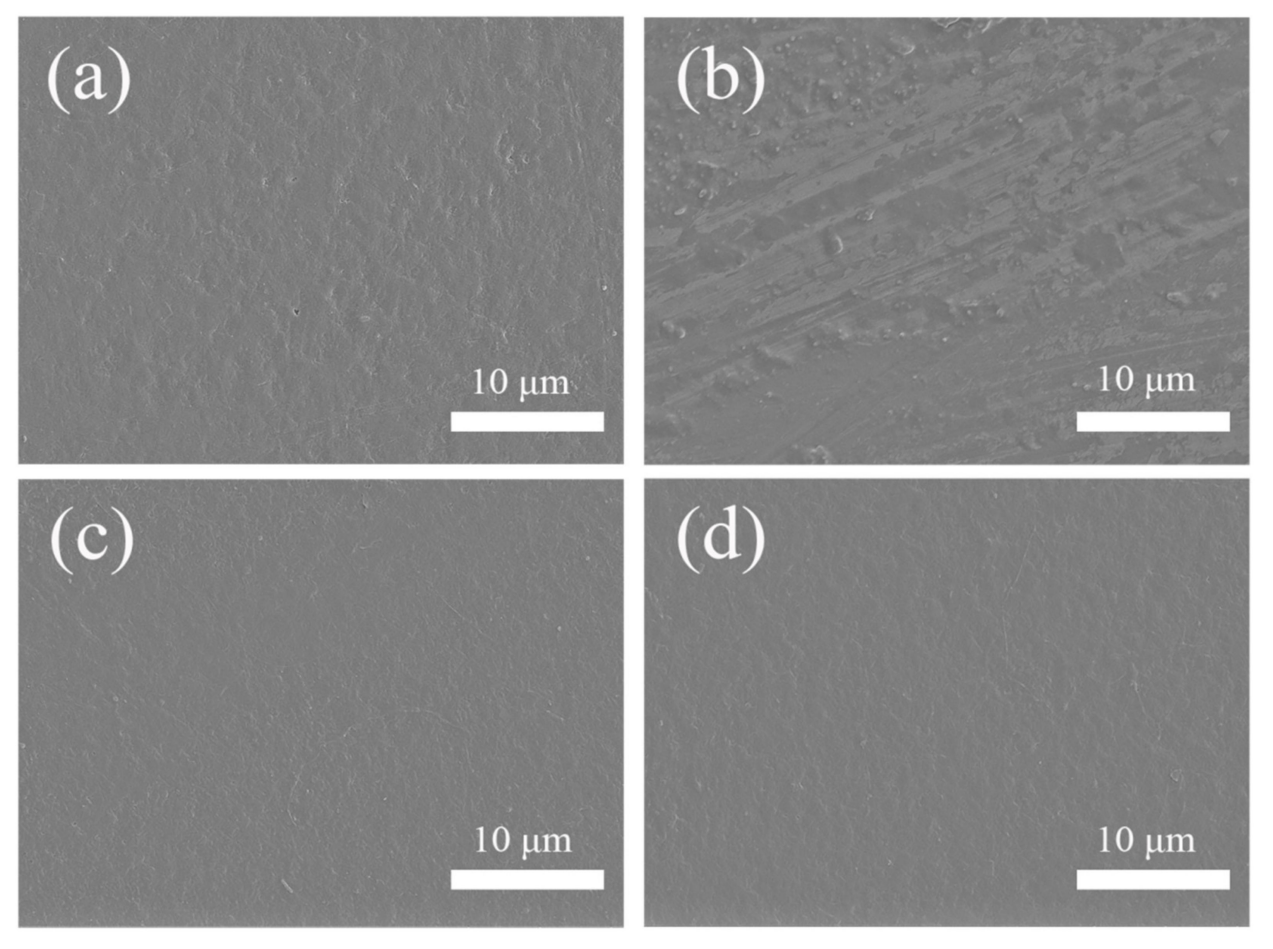
SEM images of the composites before and after corrosion treatment: (**a**,**b**) COC/20 *vol*% Sr_2_CeO_4_; (**c**,**d**) COC/20 *vol*% Sr_2_CeO_4_@SiO_2_.

To verify the corrosion resistance of the composites, the untreated and modified plates were placed in a solution of hydrochloric acid, sodium hydroxide and isopropanol for 5 min, respectively, and then the surfaces of the plates were characterized by SEM. Figure 9 shows the SEM images of the composites before and after corrosion. The surface of the modified composites remained flat and were not significantly affected. However, the surface of the untreated composites became uneven and had obvious corrosion marks. The SiO_2_ shell has good stability and can protect the composites from corrosion. The corrosion resistance of composites filled with modified fillers was significantly enhanced, which is beneficial when maintaining a better physical stability in complex and harsh environments.

### 3.5. Coefficient of Thermal Expansion and Mechanical Properties

**Figure 10:**
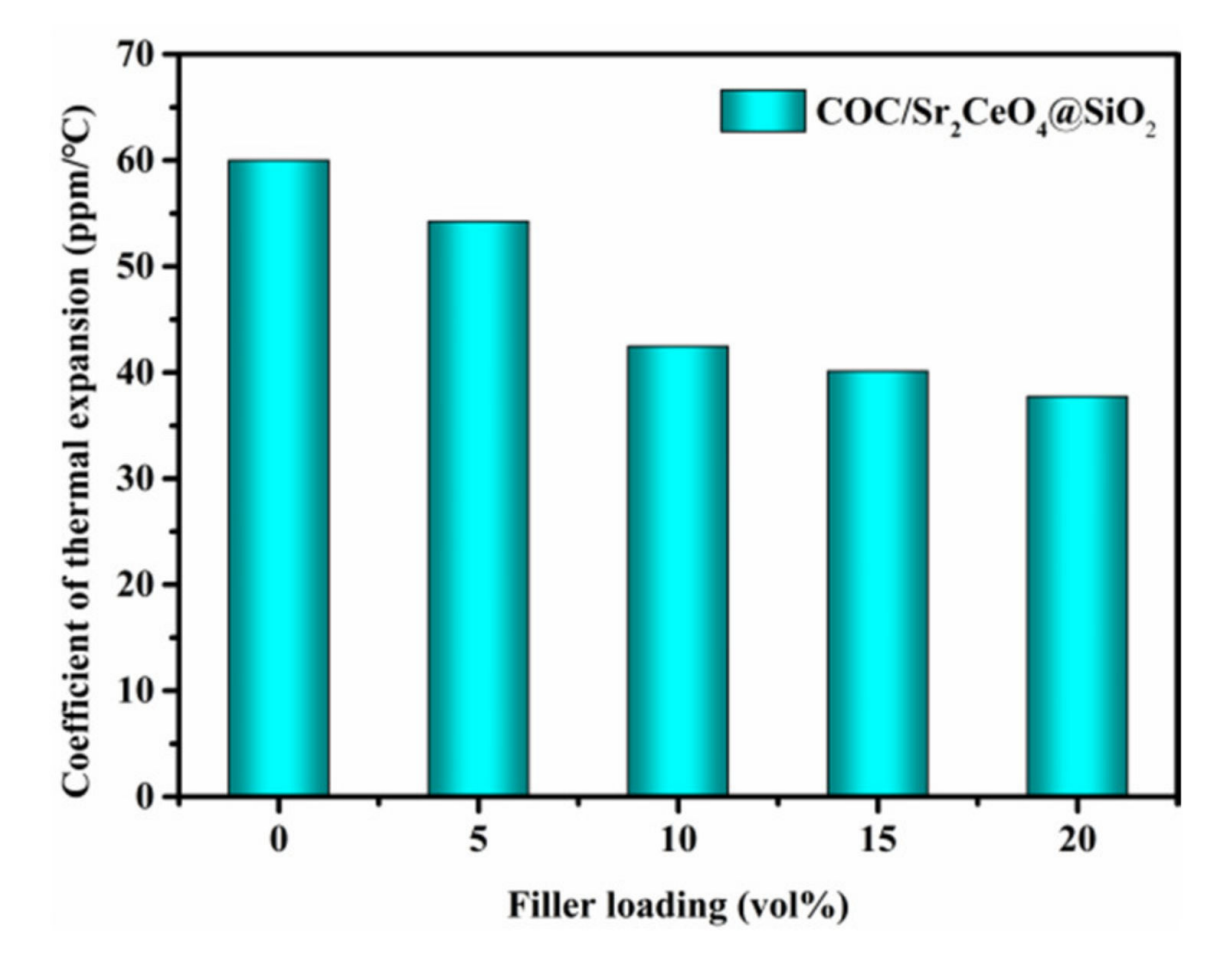
The coefficient of thermal expansion of COC/Sr_2_CeO_4_@SiO_2_ composites.

**Figure 11:**
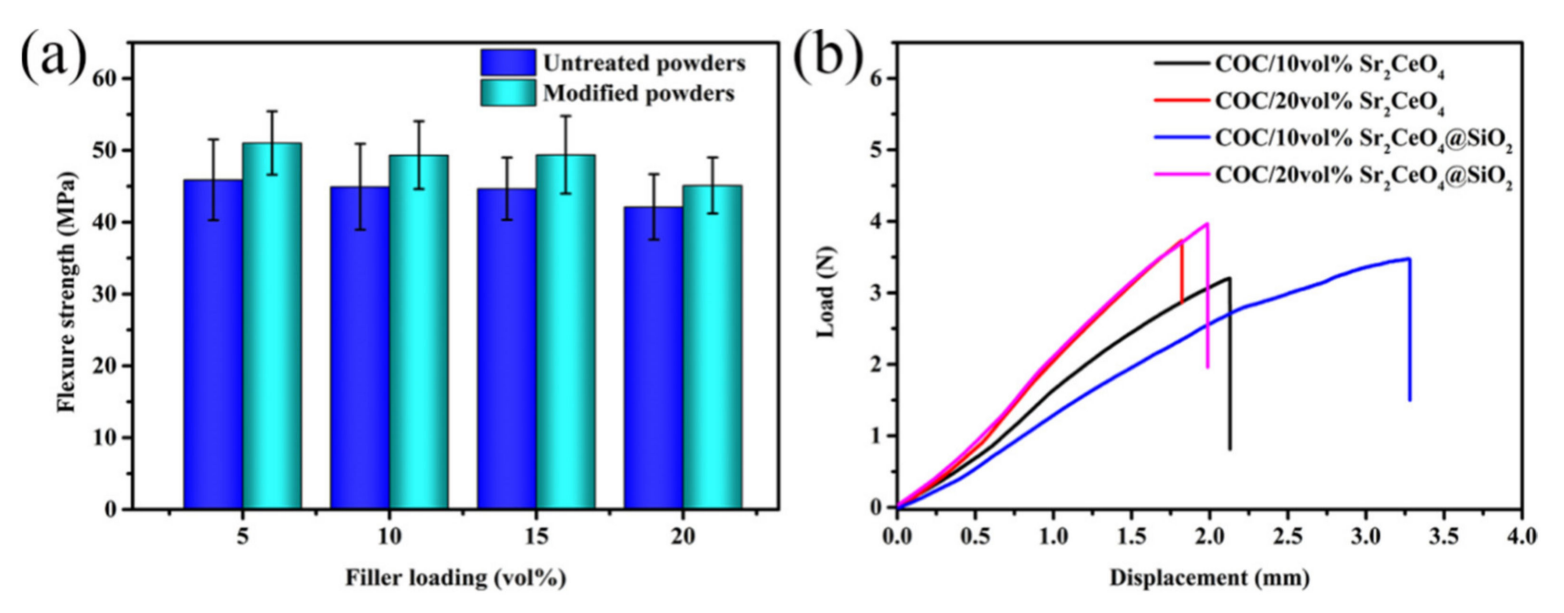
(**a**) The flexure strength of the composites; (**b**) the load-displacement curves of the composites.

Figure 10 shows the coefficient of thermal expansion (CTE) of COC/Sr_2_CeO_4_@SiO_2_ composites. The CTE of composites is affected by the type and content of fillers. With the introduction of more fillers, the CTE of the composites decreases, reaching a minimum of 37.7 ppm/°C at a filler content of 20 *vol*%. The lower coefficient of thermal expansion of the composites can reduce the thermal stress with the copper foil and improve the thermal stability of the copper-clad laminate [8]. Figure 11a shows the flexural strength of the untreated and modified composites. The flexural strength of the composites is maintained above 40 Mpa, which can be attributed to the compactness of the composites. The SiO_2_ shell can provide greater friction during the fracture process, so the flexural strength of the modified composites is higher [10]. As the filler content increases, the flexure strength of the composites decreases, which is due to the increase in internal defects of the composites. Figure 11b shows the load-displacement curves of composites. Since the thickness of the plate is only about 0.3 mm, the maximum load of the composites is relatively small. The maximum load of untreated and modified composites remains basically unchanged. However, the maximum displacement of the modified composites increases significantly. Compared with the untreated composite, the maximum displacement of the modified composite is increased by 56% at a filler content of 10 *vol*%. Through powder modification, the mechanical properties of the composites are enhanced.

Compared with other composites, the COC-based composites filled with Sr_2_CeO_4_@SiO_2_ exhibit excellent comprehensive performance (Table 2). The COC/20 *vol*% Sr_2_CeO_4_@SiO_2_ composite maintains low ε_r_ (3.5) and low τ_ε_ (0.0023) at 10 GHz, while exhibiting a thermal conductivity of 0.9 W m^−1^ K^−1^. Therefore, the COC/Sr_2_CeO_4_@SiO_2_ composites have potential application prospects in the high-frequency substrate industry.

## 4. Conclusions

A simple preparation method was reported for hydrocarbon resin-based composites. The composites exhibit dielectric properties of ε_r_ < 3.5 and τ_ε_ < 0.0023 at 10 GHz. Compared with the COC matrix, the thermal conductivity of the composites is improved by 128%, reaching 0.9 W m^−1^ K^−1^ at a filler content of 20 *vol*%. Due to the protection of the SiO_2_ shell on the filler surface, the water absorption of the composites is kept below 0.32%. The introduction of Sr_2_CeO_4_@SiO_2_ fillers can also effectively reduce the coefficient of thermal expansion to 37.7 ppm/°C and maintain the better dimensional stability of the composites. In addition, the composites exhibit improved mechanical properties, with a flexural strength above 40 Mpa. These results demonstrate that COC/Sr_2_CeO_4_@SiO_2_ composites have excellent comprehensive properties and application prospects in the high-frequency communication industry.

## Figures and Tables

**Table 1:** The chemical composition of the samples obtained by XPS method in at %.

Sample	Si 2p	O 1s	Sr 3d	Ce 3d	C 1s
Sr_2_CeO_4_	-	56.24	12.98	1.05	29.73
Sr_2_CeO_4_@SiO_2_	6.17	67.05	7.21	0.16	19.42

**Table 2:** Performance comparison of different composites.

Composites	Loading	ε_r_	τ_ε_	F (Hz)	TC (W·m^−1^·K^−1^)	Reference
PPE/BN@SiO_2_	50.0 wt.%	3.94	0.0040	5 × 10^9^	1.08	[9]
PTFE/Si_3_N_4_	62.0 *vol*%	4.03	0.0014	10^10^	1.30	[19]
SIR/BN/CNTs	12.9 *vol*%	2.90	0.0010	10^6^	0.28	[38]
SIR/BN/AlN	50.0 wt.%	3.98	0.0085	10^6^	0.56	[39]
PDMS/BNNS/BNF	10.4 wt.%	2.97	0.0050	10^9^	0.56	[40]
PAA/BN	30.0 wt.%	3.77	0.0070	10^6^	0.70	[41]
COC/Sr_2_CeO_4_@SiO_2_	20.0 *vol*%	3.50	0.0023	10^10^	0.90	This work

## Data Availability

The data presented in this study are available from the corresponding author on reasonable request.
